# PaCO_2_ as a Possible Treatable Trait in Acute Respiratory Failure: A Scoping Review

**DOI:** 10.3390/jcm15103985

**Published:** 2026-05-21

**Authors:** Carmelo Dueñas-Castell, José Correa-Guerrero, Dairo Rodelo-Barrios, Luis Valderrama-Ortiz, Cristhian Vallejo-Burgos, Diana Borré-Naranjo, Amilkar Almanza-Hurtado, Elber Osorio-Rodríguez

**Affiliations:** 1 Faculty of Medicine, Universidad Metropolitana de Barranquilla, Barranquilla 080002, Colombia; crdc2001@gmail.com; 2 Intensive Care and Obstetrics Research Group (GRICIO), University of Cartagena, Cartagena de Indias 130005, Colombia; dr.amilkar.almanza@gmail.com; 3 Department of Critical Medicine, Clínica Gestión Salud, Cartagena de Indias 130005, Colombia; 4 Department of Critical Medicine and Intensive Care, University of Cartagena, Cartagena de Indias 130005, Colombia; josegabriel2101@gmail.com; 5 Department of Critical Medicine, Hospital Universitario del Caribe, Cartagena de Indias 130005, Colombia; 6 Critical and Internal Medicine Research Group (CRITIMED), Cartagena de Indias 130005, Colombia; dairorodelo1992@gmail.com; 7 Department of Critical Medicine and Intensive Care, Faculty of Medicine, Simón Bolívar University, Barranquilla 080002, Colombia; luisvalderrama14@hotmail.com (L.V.-O.); cristian.vallejo2016@gmail.com (C.V.-B.); 8 Department of Critical Medicine, Hospital Serena del Mar, Cartagena de Indias 130005, Colombia; dianaborren0424@gmail.com; 9 Department of Intensive Medicine, Clínica Iberoamérica, Barranquilla 080002, Colombia; 10 Group Care Medicine, Clínica Colsanitas, Bogotá D.C. 110931, Colombia

**Keywords:** PaCO_2_, dyscapnia, acute respiratory failure, non-invasive ventilation, high-flow nasal cannula, mechanical ventilation, weaning, scoping review

## Abstract

Acute respiratory failure (ARF) often leads to ICU admission, ventilatory support, illness, and death. The usual classification into hypoxemic and hypercapnic types does not capture its full complexity. Precision medicine uses the concept of “treatable traits” to guide care based on traits that are clinically relevant, identifiable, measurable, and possibly changeable. Arterial carbon dioxide pressure (PaCO_2_) reflects factors like alveolar ventilation, dead space, respiratory mechanics, and how patients respond to ventilatory support. This makes it clinically relevant in selected situations. We carried out a scoping review using PRISMA-ScR and JBI guidelines to summarize evidence on hypocapnia and hypercapnia as prognostic, stratification, or clinically relevant variables during respiratory support. We searched PubMed/MEDLINE, ScienceDirect, and Web of Science (1994–2025), and checked references by hand. Thirty-four studies met our criteria and were grouped into four areas: pre-intubation or early acute presentation, non-invasive support (NIV/HFNC), invasive mechanical ventilation (IMV), and weaning or post-extubation. In summary, hypocapnia was linked to worse outcomes or failure of support in hypoxemic or cardiogenic cases. Hypercapnia helped identify patients who benefited from NIV, such as those with chronic obstructive pulmonary disease or obesity hypoventilation. For IMV, the effects depended on the presence and severity of acidosis and on its duration. Overall, PaCO_2_ showed context-dependent clinical relevance, acting mainly as a prognostic or stratification marker and, in narrower settings, as a variable that may inform monitoring or support decisions. This review provides a pragmatic framework for interpreting PaCO_2_ across respiratory support contexts and highlights the need for safe and clinically meaningful targets.

## 1. Introduction

Acute respiratory failure (ARF) is one of the most prevalent and clinically relevant syndromes in critical care medicine and represents a major cause of admission to intensive care units (ICUs), the need for ventilatory support, and significant hospital morbidity and mortality [1]. Although its classic approach has been based on the distinction between hypoxemic and hypercapnic forms, this classification offers an incomplete view of a heterogeneous, dynamic pathophysiological process that is strongly dependent on the clinical setting [2]. In this context, the growing interest in precision medicine strategies has favored the incorporation of the concept of “treatable traits,” which, in operational terms, must be clinically relevant, identifiable, measurable, and potentially modifiable through intervention [3,4,5]. Within this framework, arterial carbon dioxide pressure (PaCO_2_) emerges as a candidate of particular interest, as it integrates information on alveolar ventilation, physiological dead space, respiratory mechanics, and response to ventilatory support. Furthermore, it may be involved in biological pathways with potential immunomodulatory relevance, lending mechanistic plausibility to its consideration as a potentially treatable trait in selected scenarios of ARF [6,7].

Recent clinical evidence suggests that PaCO_2_ alterations, including hypocapnia and hypercapnia, are associated with clinically important outcomes across the respiratory support continuum, from non-invasive ventilation (NIV) to invasive mechanical ventilation (IMV) and the weaning or post-extubation phase [8,9,10,11,12,13]. In this regard, PaCO_2_ could provide valuable information not only as a physiological marker, but also as a variable with potential utility for clinical stratification and therapeutic guidance in selected ARF scenarios [6]. Additionally, particularly complex situations, such as refractory hypercapnia, illustrate both the complexity of ventilatory management and the need for operational physiological frameworks to guide decision-making [14]. However, the available evidence remains heterogeneous in terms of the populations studied, clinical contexts, and operational definitions of PaCO_2_ alterations, which limits an integrated understanding of its role as a potential treatable trait in ARF.

In this context, we conducted a scoping review with the aim of mapping the available evidence and identifying knowledge gaps regarding the role of PaCO_2_ as a potential treatable trait in adults with ARF, with particular emphasis on the clinical scenarios in which it has been studied (pre-intubation, NIV/high-flow nasal cannula [HFNC], IMV, and weaning/post-extubation), identifying its association with relevant outcomes and its potential usefulness for prognostic stratification and therapeutic decision-making [15,16]. Therefore, the question that guided this review was as follows: What evidence is there on the role of PaCO_2_ as a possible treatable trait in adults with ARF, and what gaps remain for its use in risk stratification and therapeutic decision-making?

## 2. Materials and Methods

We developed and reported this scoping review according to the PRISMA-ScR guidelines (Preferred Reporting Items for Systematic Reviews and Meta-Analyses extension for Scoping Reviews) [15,16] and followed methodological recommendations for scoping reviews. We registered the protocol on the Open Science Framework (OSF; https://osf.io/vszkg) on 18 March 2026. Because registration took place after the study period and initial strategy were set, it was considered post hoc. We documented any later changes in a decision log (Supplementary Table S1). The PRISMA-ScR checklist is available in Supplementary Table S2.

### 2.1. Eligibility Criteria

This scoping review was structured according to the PCC framework (Population, Concept, Context), as recommended for JBI scoping reviews. The population comprised adult patients with ARF or acute respiratory deterioration; the concept was the clinical interpretation of PaCO_2_, hypercapnia, hypocapnia, or related CO_2_ derangements as exposure, stratification, monitoring, or prognostic variables; and the context included pre-intubation, non-invasive respiratory support, IMV, and weaning/post-extubation settings.

Original studies in adult patients with ARF or acute respiratory deterioration were included, in which PaCO_2_, hypercapnia, hypocapnia, or CO_2_ dysfunction were assessed as exposure, stratification, monitoring, or prognostic variables. Prospective or retrospective observational studies, randomized controlled trials, and comparative studies reporting clinically relevant outcomes, including mortality, non-invasive ventilation failure, need for intubation, duration of mechanical ventilation, extubation failure, reintubation, or length of hospital stay, were considered. Studies published in English or Spanish were considered eligible. We excluded studies in pediatric populations, animal models, narrative reviews, editorials, letters to the editor, and publications without sufficient clinical data for extraction. Studies focused exclusively on stable chronic populations without acute exacerbations; episodes of acute respiratory failure were also excluded.

### 2.2. Information Sources and Search Strategy

A structured literature search was conducted in PubMed/MEDLINE, ScienceDirect, and Web of Science to identify studies on PaCO_2_ alterations across the clinical continuum of acute respiratory failure. The strategy combined controlled terms and free-text words related to “PaCO_2_,” “hypercapnia,” “hypocapnia,” “dyscapnia,” “hypercapnic acidosis,” “acute respiratory failure,” “non-invasive ventilation,” “high-flow nasal cannula,” “mechanical ventilation,” “weaning,” and “extubation.” The complete strategy is reported in Supplementary Table S3. Additionally, a manual search of references was performed using the selected articles to identify potentially relevant studies not captured in the electronic search. Duplicates were managed in EndNote X8.

### 2.3. Study Selection

The retrieved records were exported to a reference manager for duplicate removal. Subsequently, the selection was carried out in two phases: (i) screening by title and abstract according to the previously defined eligibility criteria and (ii) full-text review of potentially relevant studies. Screening was conducted independently by two reviewers. Disagreements were resolved by discussion and, when necessary, by consultation with a third reviewer. Articles that explicitly addressed the role of PaCO_2_ in any of the scenarios defined for mapping were ultimately included early acute presentation/pre-intubation, non-invasive respiratory support, invasive mechanical ventilation, ventilator weaning, or the post-extubation period.

### 2.4. Data Extraction

Data extraction was performed independently by two reviewers. Extracted variables included author and year, study design, sample size, population and clinical setting, operational definitions of hypercapnia/hypocapnia (including cut-off values when available), primary objective, and relevant outcomes (mortality, treatment failure, intubation, duration of mechanical ventilation, weaning, extubation/reintubation, and length of stay). The role assigned to PaCO_2_ in each study was also recorded (prognostic marker, physiological monitoring variable, stratification criterion, or context-dependent variable with potential clinical relevance). Discrepancies in extracted data were resolved by consensus, with adjudication by a third reviewer when required.

### 2.5. Evidence Synthesis and Mapping

Given the exploratory nature of this scoping review and the clinical and methodological heterogeneity of the included studies, a meta-analysis was not performed. The evidence was synthesized narratively and organized into predefined clinical domains: (1) pre-intubation/early acute presentation, (2) non-invasive respiratory support, (3) invasive mechanical ventilation, and (4) weaning/post-extubation. These domains were used as practical categories for organizing the evidence, rather than as a fixed sequence of progressively more invasive therapies. In clinical practice, patients do not always follow the same pathway and may move between these settings depending on the underlying cause, severity, response to treatment, and local management strategies. This approach allowed us to describe how PaCO_2_ has been evaluated in different clinical settings and to identify patterns of association between hypocapnia, hypercapnia, and relevant outcomes.

### 2.6. Transparency and Data Availability

All analyzed data were obtained from previously published studies accessible in the consulted databases. The search strategy, selection criteria, and extraction variables were explicitly defined to promote reproducibility. We deposited the decision log (Supplementary Table S1), the PRISMA-ScR checklist, and the complete search strategies (Supplementary Tables S2 and S3) on OSF.

## 3. Results

**Figure 1:**
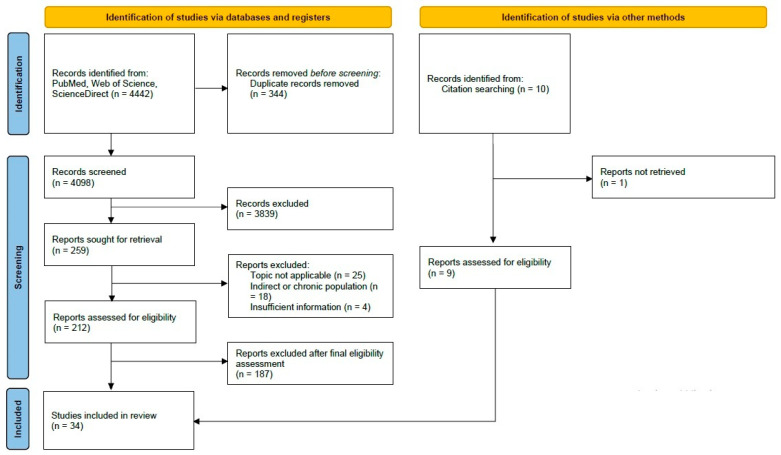
PRISMA-ScR flow diagram.

The electronic search found 4442 records. After removing 344 duplicates, 4098 unique references remained for the initial screening. Reviewing titles and abstracts excluded 3839 records. We then evaluated 259 full texts and excluded 234 that did not address the research question. In the end, 25 studies were chosen from the primary search, and 9 more were added from the secondary search. Altogether, 34 studies met the inclusion criteria (Figure 1). Table 1 summarizes the general characteristics, including author, year, country, and study design.

### 3.1. Characteristics of the Included Studies

The 34 included studies, published between 1994 and 2025, evaluated the clinical implications of PaCO_2_ alterations across different stages of acute respiratory failure and during respiratory support. Sample sizes ranged from 13 to 252,812 participants. Collectively, the included studies comprised 268,123 patients, excluding one study for which the sample size was not available in the preliminary extraction table. Most studies were observational (*n* = 27), including retrospective and prospective cohorts, while 7 were randomized controlled trials.

The evidence is distributed across four main clinical domains: pre-intubation phase or early acute presentation (*n* = 4), non-invasive respiratory support including NIV and HFNC (*n* = 12), IMV and assessment of CO_2_ dysfunction in the ICU (*n* = 9), and ventilatory weaning or post-extubation management (*n* = 9).

### 3.2. Mapping of Evidence by Clinical Domain

Table 2 maps the included studies according to the main clinical context in which PaCO_2_ was assessed. These domains are descriptive and may overlap in real-world practice.

#### 3.2.1. Pre-Intubation and Early Acute Presentation

In this review, the pre-intubation and early acute presentation domain refers to patients evaluated during the initial phase of acute respiratory failure, before IMV when applicable, or during early clinical deterioration. Four studies looked at how changes in PaCO_2_ affect patients during the early stages of ARF or acute cardiopulmonary decompensation [8,9,10,44]. In these studies, low PaCO_2_, or hypocapnia, often signaled worse outcomes. For patients with acute heart failure, cardiogenic acute pulmonary edema (APE), hypoxemic respiratory failure, and cardiogenic shock, lower PaCO_2_ levels were linked to higher mortality [8,44] and, in some groups, a greater risk of non-invasive ventilation failure [9,10]. Overall, these findings suggest that hypocapnia may help identify patients with a more severe form of acute respiratory failure early on. See Table 2.

#### 3.2.2. Non-Invasive Respiratory Support

The non-invasive respiratory support domain includes studies in which NIV or HFNC constituted the main support strategy being evaluated, regardless of whether patients had received prior conventional oxygen therapy or other initial measures. Twelve studies evaluated PaCO_2_ in the context of NIV or HFNC, primarily in patients with acute exacerbations of chronic obstructive pulmonary disease (COPD), obesity hypoventilation syndrome, or APE [20,29]. In acute hypercapnic respiratory failure, NIV was consistently associated with favorable outcomes, including reduced intubation rates and high therapeutic success rates in several observational cohorts and clinical trials [20,29]. In contrast, studies in hypercapnic COPD have evaluated alternatives, such as HFNC, compared with standard strategies, suggesting variable efficacy depending on severity and context [37]. Taken together, these studies support the clinical relevance of hypercapnia as a marker of a phenotype in which non-invasive ventilatory support may be particularly beneficial. See Table 2.

#### 3.2.3. Invasive Mechanical Ventilation and CO_2_ Dysfunction in the ICU

Nine studies evaluated the association between PaCO_2_ alterations and outcomes in patients undergoing IMV, including cohorts with ARDS, COVID-19, acute brain injury, and general ICU populations [11,12,38,41]. In this domain, the relationship between PaCO_2_ and outcomes varied by clinical context. Several studies have shown that both hypocapnia and marked hypercapnia are associated with worse outcomes, including increased mortality, ventilator load, and prolonged IMV [12,41]. Hypercapnic acidosis during the first 24–48 h of mechanical ventilation appears to be particularly consistently associated with adverse outcomes [11]. However, not all studies demonstrated excess mortality associated with hypercapnia, suggesting that the prognostic impact of elevated PaCO_2_ may depend on its magnitude, temporal profile, degree of compensation, and underlying clinical phenotype [38]. See Table 2.

#### 3.2.4. Ventilatory Weaning and Post-Extubation

The weaning and post-extubation domain includes both predictors of weaning or extubation failure and studies evaluating post-extubation support strategies, including reintubation as a clinically relevant outcome when reported. Nine studies focused on the role of PaCO_2_ during weaning from mechanical ventilation and the post-extubation period [13,28]. In this context, hypercapnia was associated with a risk of post-extubation respiratory failure and more complex weaning trajectories, supporting preventive or rescue strategies with non-invasive support [13]. Furthermore, another study reported that NIV reduced post-extubation respiratory failure and reintubation in selected hypercapnic patients [13]. These findings suggest that PaCO_2_ not only functions as a physiological marker in the post-extubation phase but also as a clinically actionable variable for risk stratification and optimization of respiratory support strategies. See Table 2.

## 4. Discussion

### 4.1. PaCO_2_ in Hypoxemic Respiratory Failure

In patients with hypoxemic respiratory failure, PaCO_2_ should not be interpreted as a defining diagnostic criterion, but rather as a contextual physiological variable. In this setting, low or low–normal PaCO_2_ values may reflect increased respiratory drive, greater ventilatory demand, or evolving respiratory distress [10,45]. Recent guidelines and meta-analyses support the early use of HFNC over conventional oxygen therapy and NIV in selected forms of acute hypoxemic respiratory failure [46,47]. In APE, recent evidence also supports the use of HFNC [48,49]; however, current guidelines continue to recommend NIV as the first-line strategy in this scenario [50,51,52]. In this population, PaCO_2_ appears to function primarily as a prognostic marker. Consequently, in patients with acute heart failure/APE, a PaCO_2_ < 31 mmHg was associated with increased mortality, and the risk increased as PaCO_2_ decreased [8]. Hypocapnia has also been consistently linked to NIV failure and an increased risk of mortality in patients with APE [9].

**Figure 2:**
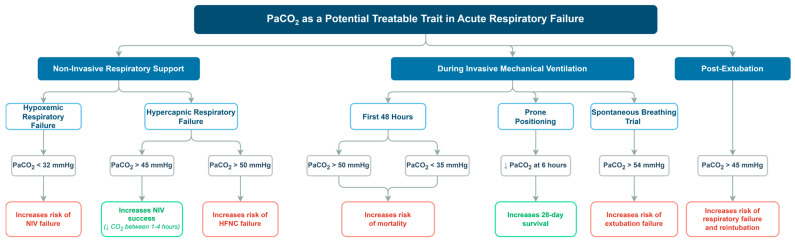
Proposed conceptual framework for the clinical interpretation of PaCO_2_ in acute respiratory failure. The figure summarizes the context-dependent associations identified across the included studies. Similar PaCO_2_ values may have different clinical implications depending on the respiratory support strategy, timing of assessment, acid–base status, and stage of care. In most scenarios, PaCO_2_ functions primarily as a prognostic or stratification marker; only in selected contexts may it help support modality selection or monitoring of response. The figure is intended as a conceptual summary rather than a treatment algorithm. Created by the authors.

Beyond APE, an association has been described between PaCO_2_ ≤ 32 mmHg and NIV failure in hypoxemic respiratory failure, with an inverse relationship between PaCO_2_ and the risk of failure above this threshold [10]. From a physiological perspective, hypocapnia may reflect increased minute ventilation and/or elevated tidal volumes, which are variables associated with NIV failure in de novo hypoxemia [53], and may indicate increased respiratory drive, vigorous inspiratory effort, and marked negative oscillations in pleural pressure [54]. These mechanisms may promote self-induced lung injury (SILI), as supported by experimental evidence showing that intense spontaneous exertion is associated with elevated transpulmonary pressures and worsening lung damage [55]. Overall, in acute hypoxemia, PaCO_2_ is a readily available and clinically relevant signal that helps identify a high-exertion phenotype and a higher risk of non-invasive support failure. See Figure 2.

### 4.2. PaCO_2_ in Hypercapnic Respiratory Failure

In hypercapnic respiratory failure (pH ≤ 7.35 and PaCO_2_ > 45 mmHg), evidence supports NIV as a first-line intervention [52,56]. In APE, hypercapnia is relatively common in a clinically relevant subgroup of patients and may help identify individuals more likely to require or benefit from NIV; however, APE itself should not be equated with hypercapnic respiratory failure. In this subgroup, NIV has been associated with a lower need for intubation and lower in-hospital mortality [9], and randomized evidence supports its efficacy in APE [22]. Furthermore, in patients with pulmonary edema treated with NIV, severe hypercapnia (PaCO_2_ > 60 mmHg) has been associated with a longer duration of NIV, without a proportional increase in the risk of intubation [57].

In other etiologies, hypercapnia accompanied by severe acidosis may still warrant NIV and may be successfully managed in selected patients, including those treated in intermediate respiratory care units [58]. Some observational studies have also reported the use of NIV in selected cases of hypercapnic encephalopathy or even hypercapnic coma [23,59]. However, these findings should be interpreted with caution, as impaired consciousness may compromise airway protection and increase aspiration risk; therefore, intubation remains necessary when airway safety cannot be assured. Similarly, in acute hypercapnic respiratory failure associated with obesity and alveolar hypoventilation, NIV has shown clinical benefit [29], and PaCO_2_ remains a useful marker to support clinical assessment and modality selection according to the underlying cause and overall patient status.

Although there is growing interest in HFNC in hypercapnic respiratory failure, comparative literature suggests that NIV remains the gold standard, and meta-analytic evidence comparing HFNC vs. NIV in acute hypercapnic respiratory failure supports careful patient selection [60]. In line with this, a randomized trial in acute COPD exacerbation showed a higher progression rate to IMV with HFNC than to NIV in acute-to-moderate hypercapnic respiratory failure [42]. After initiating NIV, the early trajectory of PaCO_2_ provides useful clinical information: a decrease is a favorable predictor of success and may support weaning, whereas the absence of early improvement suggests a higher risk of failure. Overall, in hypercapnic respiratory failure, PaCO_2_ is identifiable and measurable, and in selected hypercapnic phenotypes it may help support modality selection; however, its direct treatability remains context-dependent. See Figure 2.

### 4.3. PaCO_2_ in Patients on Invasive Mechanical Ventilation

Once the patient is intubated, PaCO_2_ remains a key variable for monitoring and prognosis. A large, multicenter, retrospective study (252,812 patients) found that hypercapnic acidosis (PaCO_2_ > 45 mmHg; pH < 7.35) in the first 24 h was more strongly associated with higher in-hospital mortality than compensated hypercapnia or normocapnia [11]. In moderate-to-severe ARDS, marked hypercapnia during the first 48 h has also been associated with higher ICU mortality [12]. Importantly, in patients with COVID-19 on IMV, hypercapnia was associated with longer ventilation duration and longer ICU/hospital stays [39]. However, the clinical impact of a given PaCO_2_ level depends on the underlying mechanism and the ventilation strategy. A meta-analysis in ARDS assessed acute hypercapnia and its relationship to short-term mortality and physiology [61], and early changes in PaCO_2_ have also been proposed as predictors of prolonged ventilation [43]. In parallel, integrating variables such as mechanical power and ventilator ratio have been associated with mortality during IMV [62,63,64]. Furthermore, dyscapnia has been associated with mortality in ARDS cohorts [40], and the LUNG SAFE study showed higher ICU mortality in patients with sustained hypocapnia in mild-to-moderate ARDS [38]. In cardiogenic shock, hypocapnia has consistently been associated with increased 30-day mortality [44].

In neurocritical patients, prophylactic hyperventilation to very low PaCO_2_ levels is discouraged in modern head trauma guidelines [65]. In acute kidney injury under mechanical ventilation, both severe hypocapnia and hypercapnia were associated with increased in-hospital mortality [41], reinforcing the notion of “risk zones” at the extremes of PCO_2_. On the other hand, prone positioning is an intervention with strong recommendations for ARDS [66,67]. In a retrospective analysis, responders to prone positioning, defined as a PaCO_2_ decrease ≥ 1 mmHg after 6 h, showed improved 28-day survival [21]. Physiological studies suggest that PaCO_2_ and dead space metrics may better capture the response to prone positioning than oxygenation alone [68]. See Figure 2.

### 4.4. PaCO_2_ in Extubation and Post-Extubation Outcomes

Finally, in the weaning and post-extubation phase, hypercapnia during the spontaneous breathing trial has been associated with prolonged weaning and extubation failure [27,31]. Following extubation, NIV has shown efficacy in preventing post-extubation respiratory failure and intubation in hypercapnic patients [13,28,69]. In high-risk patients, HFNC combined with NIV has also been evaluated against HFNC alone; it was associated with a reduction in reintubation [36]. This effect appears to be concentrated in high-risk populations, so its extrapolation to low-risk patients should be approached with caution. Regarding advanced therapies, extracorporeal CO_2_ removal has been evaluated as a strategy to correct severe hypercapnia and facilitate protective ventilation [70], and its evidence has been synthesized in recent meta-analyses [71,72], although its clinical role still requires confirmation. See Figure 2.

Taken together, the evidence suggests that the interpretation of PaCO_2_ is highly context- and mechanism-dependent: in acute hypoxemia, hypocapnia often reflects a high respiratory drive phenotype and a greater risk of non-invasive support failure; in hypercapnic failure due to COPD/obesity–hypoventilation, hypercapnia identifies a phenotype in which NIV is particularly effective and where early PaCO_2_ changes signal a response. In IMV, both sustained hypocapnia and hypercapnia accompanied by acidosis have been associated with worse outcomes in various settings, suggesting the need to define safety margins based on pH and exposure time. Thus, rather than a “universal target,” PaCO_2_ can be conceptualized as a potential stratification variable whose clinical utility lies in integrating physiology, prognosis, and strategy selection along the continuum of ventilatory failure.

Table 3 summarizes the main clinical contexts in which PaCO_2_ may have prognostic value, support monitoring, or help inform respiratory support decisions in selected patients.

### 4.5. Limitations

Several limitations must be considered when interpreting the findings. First, as a scoping review, this study was designed to map the extent and characteristics of the available evidence rather than to estimate effect sizes, establish causality, or determine whether targeted modification of PaCO_2_ improves patient-centered outcomes. In addition, although a structured search strategy was applied in indexed databases (PubMed/MEDLINE, ScienceDirect, and Web of Science) and supplemented by manual reference review, some relevant studies may have been missed, particularly in gray literature, conference abstracts, non-indexed publications, or when PaCO_2_ was embedded within related physiological constructs rather than explicitly indexed as a primary variable. Because the search strategy was intentionally centered on PaCO_2_ and direct dyscapnia-related terminology, studies focused on related constructs such as dead space, ventilatory ratio, minute ventilation, or non-invasive ventilation failure may not have been captured.

Second, the included evidence was markedly heterogeneous across populations, clinical settings, respiratory support modalities, timing of PaCO_2_ assessment, operational definitions, and outcomes. Most studies were observational, and many were retrospective and single-center, increasing susceptibility to selection bias, residual confounding, and limited external validity. A formal risk-of-bias assessment was not performed, in keeping with the exploratory purpose of a scoping review. Therefore, the observed associations between PaCO_2_ alterations and clinical outcomes should be interpreted cautiously and should not, by themselves, be considered sufficient to justify PaCO_2_-targeted practice changes across ARF scenarios.

### 4.6. Clinical Implications and Future Directions

Taken together, current evidence suggests that PaCO_2_ is a clinically informative, context-dependent physiological variable in ARF. Its main value appears to lie in prognostic assessment, early evaluation of treatment response, and support for clinical stratification in scenarios such as non-invasive ventilation, weaning, extubation, and invasive mechanical ventilation. However, substantial heterogeneity across populations, cut-off values, timing of measurement, and reported outcomes limits the development of standardized clinical algorithms. Future prospective studies should evaluate PaCO_2_-defined subgroups, temporal trajectories, and intervention thresholds to determine whether PaCO_2_ can be used safely and effectively beyond its current role as a marker of physiological stress and risk. At present, the available evidence supports PaCO_2_ primarily as a prognostic and stratification variable rather than as an independent therapeutic target. Accordingly, PaCO_2_ abnormalities should not be interpreted as causal or, by themselves, sufficient to justify protocolized treatment changes across ARF scenarios. Another underreported dimension in the available literature is right ventricular function, which may have important prognostic and pathophysiological relevance in acute respiratory failure but was not consistently described across the included studies and should be better addressed in future research.

## 5. Conclusions

The clinical relevance of PaCO_2_ varies across the continuum of acute respiratory failure. In the early presentation or pre-intubation phase, hypocapnia may identify patients with increased respiratory drive and a greater risk of non-invasive support failure. During non-invasive respiratory support, hypercapnia helps define phenotypes, particularly chronic obstructive pulmonary disease and obesity hypoventilation syndrome, in which non-invasive ventilation is most likely to be beneficial, while early changes in PaCO_2_ may support response assessment. In intubated patients, the interpretation of PaCO_2_ is more complex and depends on pH, timing, and the underlying pathophysiology rather than on a single numerical target. This relevance extends to the weaning and post-extubation phases, where hypercapnia may help identify patients at higher risk of extubation failure and support the use of non-invasive post-extubation strategies in selected settings. Overall, PaCO_2_ is best understood as a context-dependent clinical marker rather than a universal therapeutic target. This review adds to current knowledge by providing a pragmatic framework for interpreting PaCO_2_ across different respiratory support contexts and by distinguishing its prognostic role from its more selective use in clinical stratification. At the same time, as a scoping review, it is intended to map the available evidence rather than establish causal effects or define definitive treatment thresholds. Future studies should define safe and clinically meaningful targets according to pH, dyscapnia severity, and exposure time.

## Figures and Tables

**Table 1:** General characteristics of included studies (chronological order by year of publication).

Citation	Year	Country	Type of Study
Piper AJ [17]	1994	Australia	Retrospective observational study
Rabec C [18]	1998	France	Prospective observational study
Hilbert G [19]	1998	France	Observational case–control study
Plant PK [20]	2000	United Kingdom	Multicenter randomized controlled trial
Gattinoni L [21]	2003	Italy	Retrospective analysis of a randomized controlled trial
Nava S [22]	2003	Italy	Multicenter randomized controlled trial
Diaz GG [23]	2005	Spain	Prospective, open-label, uncontrolled observational study
Ferrer M [24]	2006	Spain	Prospective observational study
Ortega González [25]	2006	Spain	Prospective observational study
Duarte AG [26]	2007	USA	Retrospective observational study
Ferrer M [13]	2009	Spain	Randomized clinical trial
Sellarés J [27]	2011	Spain	Prospective cohort study
Girault C [28]	2011	France	Randomized clinical trial (multicenter)
Carrillo A [29]	2012	Spain	Prospective observational study
Marik PE [30]	2013	USA	Retrospective observational study
Pu L [31]	2015	China	Multicenter prospective cohort study
Tiruvoipati R [11]	2017	Australia/New Zealand	Retrospective multicenter observational study
Nin N [12]	2017	Multinational *	Prospective cohort study
Çiftci F [32]	2017	Turkey	Prospective observational study
Fuller BM [33]	2017	USA	Prospective observational study
Sellarés J [34]	2017	Spain	Randomized controlled trial
Bry C [35]	2018	France	Retrospective observational study
Thille AW [36]	2019	France	Randomized clinical trial
Li X [37]	2020	China	Randomized clinical trial
Madotto F [38]	2020	Multinational *	Multicenter observational study
Kato T [8]	2021	Japan	Retrospective observational study
Carrillo-Alemán L [9]	2022	Spain	Retrospective observational study
Tsonas AM [39]	2022	Netherlands	Retrospective multicenter observational study
Xu X [10]	2024	China	Retrospective observational study
Braunsteiner J [40]	2024	Germany	Retrospective observational study
Robba C [41]	2024	Multinational *	Multicenter prospective observational study
Tan D [42]	2024	China	Randomized controlled trial
Villar J [43]	2024	Multinational *	Prospective cohort study
Rusnak J [44]	2025	Germany	Prospective observational study

* Multinational indicates studies conducted across multiple countries/intensive care units.

**Table 2:** Evidence mapping by clinical domain.

Citation	Design	*n*	Context	PaCO_2_ Definition/Strata	Key Outcome
Pre-intubation/early acute presentation (before IMV initiation or in early acute decompensation)					
Kato T et al., 2021 [8]	Retrospective observational	435	Acute heart failure	PaCO_2_ as continuous; cut-offs 31 and 40 mmHg	PaCO_2_ < 31 associated with higher mortality (HR 1.71)
Carrillo-Alemán L et al., 2022 [9]	Retrospective observational	1138	Cardiogenic pulmonary edema (NIV)	Hypocapnia/eucapnia/hypercapnia (hypercapnia threshold NR)	Hypocapnia: higher NIV failure and in-hospital mortality
Xu X et al., 2024 [10]	Retrospective observational	1029	Hypoxemic respiratory failure (NIV)	Hypocapnia ≤ 32 mmHg	Hypocapnia: higher NIV failure (adjusted HR 1.23)
Rusnak J et al., 2025 [44]	Prospective observational	238	Cardiogenic shock	Hypocapnia ≤ 33 mmHg; hypercapnia > 48.13 mmHg	Hypocapnia: higher 30-day mortality; hypercapnia not associated
Non-invasive respiratory support (NIV/HFNC-treated populations)					
NIV in hypercapnic acute respiratory failure (COPD/OHS/OSA–OHS)					
Piper AJ et al., 1994 [17]	Retrospective observational	13	Obesity, BMI > 35; nocturnal nasal ventilation	PaCO_2_ > 45 mmHg	NIV success ~64–69%
Rabec C et al., 1998 [18]	Prospective observational	41	Sleep apnea + respiratory acidosis (NIV)	pH < 7.35; PaCO_2_ > 45 mmHg	Intubation avoided in 95%
Diaz GG et al., 2005 [23]	Prospective observational (open-label)	681	Hypercapnic coma (NIV)	PaCO_2_ > 45 mmHg	NIV success 80%
Ortega González et al., 2006 [25]	Prospective observational (open-label)	53	COPD, OHS, AHF (NIV)	pH > 7.25; PaCO_2_ > 45 mmHg	At 3 h: pH ↑ and PaCO_2_ ↓
Duarte AG et al., 2007 [26]	Retrospective observational	50	Morbid obesity, BMI > 35 kg/m^2^ (NIV)	PaCO_2_ > 50 mmHg	NIV success 64%
Çiftci F et al., 2007 [32]	Prospective observational	106	Hypercapnic respiratory failure (assured volume PS)	pH < 7.35; PaCO_2_ > 45 mmHg	NIV success 76.4%
Carrillo A et al., 2012 [29]	Prospective observational	716	ARF episodes due to OHS and COPD	pH < 7.35; PaCO_2_ > 45 mmHg	NIV success 88.4%
Marik PE et al., 2013 [30]	Prospective observational	61	OHS, BMI > 40 kg/m^2^ (BiPAP)	PaCO_2_ > 45 mmHg	37.7% progressed to IMV
Sellarés J et al., 2017 [34]	Randomized clinical trial	120	NIV prolongation after ARF resolution	pH < 7.35; PaCO_2_ > 45 mmHg	ARF recurrence 13%
Bry C et al., 2018 [35]	Retrospective observational	53	BMI > 30; long-term NIV after ARF hospitalization	PaCO_2_ > 45 mmHg	NIV success 90%
HFNC vs. NIV trials/strategies in hypercapnic COPD					
Plant PK et al., 2000 [20]	Multicenter randomized clinical trial	236	AECOPD: NIV	pH 7.25–7.35; PaCO_2_ > 45 mmHg	15% progressed to IMV
Li X et al., 2020 [37]	Randomized clinical trial	320	COPD: HFNC	pH > 7.35; PaCO_2_ > 45 mmHg	19% progressed to IMV
Tan D et al., 2024 [42]	Randomized clinical trial	225	COPD: HFNC vs. NIV	pH 7.25–7.35; PaCO_2_ > 50 mmHg	IMV: 25.7% (HFNC) vs. 14.3% (NIV)
IMV/ICU CO_2_ derangements					
Gattinoni L et al., 2003 [21]	Retrospective analysis of an RCT	225	ALI/ARDS (prone)	Prone responders: PaCO_2_ decrease ≥ 1 mmHg after 6 h	Responders had higher 28-day survival
Tiruvoipati R et al., 2017 [11]	Retrospective multicenter observational	252,812	IMV patients	Hypercapnia > 45 mmHg; hypercapnic acidosis: pH < 7.35	Hypercapnia/hypercapnic acidosis associated with higher mortality
Nin N et al., 2017 [12]	Prospective non-interventional cohort	889	ARDS <48 h	Hypercapnia > 40 mmHg; >50 mmHg emphasized	PaCO_2_ > 50 is associated with higher mortality (first 48 h)
Fuller BM et al., 2017 [33]	Prospective observational	1491	IMV (first 48 h)	Hypocapnia < 35 mmHg; hypercapnia > 45 mmHg	Higher survival with hypercapnia vs. hypocapnia
Madotto F et al., 2020 [38]	Multicenter observational	2813	Early ARDS	Hypocapnia < 35 mmHg; hypercapnia > 45 mmHg	Mortality: hypercapnia 36%; hypocapnia 38.1%
Tsonas AM et al., 2022 [39]	Retrospective multicenter observational	824	COVID-19 on IMV	Hypercapnia > 45 mmHg	Longer MV duration and ICU/hospital LOS
Braunsteiner J et al., 2024 [40]	Retrospective observational	435	Mechanical power + dyscapnia	Hypocapnia < 35 mmHg; hypercapnia > 50 mmHg	Hypocapnia is associated with higher mortality; hypercapnia > 50 is not associated
Robba C et al., 2024 [41]	Multicenter prospective observational	1476	Acute brain injury	Hypocapnia < 35 mmHg; hypercapnia > 45 mmHg	Both hypo- and hypercapnia associated with higher mortality
Villar J et al., 2024 [43]	Prospective cohort	253	Moderate-to-severe ARDS	Hypercapnia > 45 mmHg; early PaCO_2_ changes	Early PaCO_2_ changes predict MV duration > 14 days
Weaning, extubation, and post-extubation					
Predictors of prolonged weaning/extubation failure					
Ferrer M et al., 2006 [24]	Prospective observational	162	Weaning	Hypercapnia > 45 mmHg	Higher orotracheal re-intubation
Sellarés J et al., 2011 [27]	Prospective cohort	181	SBT/weaning	Hypercapnia > 45 mmHg; ≥54 mmHg	Associated with prolonged weaning and extubation failure
Pu L et al., 2015 [31]	Multicenter prospective cohort	343	First SBT	Hypercapnia > 50 mmHg	Associated with prolonged mechanical ventilation
Post-extubation interventions (NIV/HFNC) in hypercapnic patients					
Hilbert G et al., 1998 [19]	Case–control observational	30	COPD post-extubation	PaCO_2_ ↑ 20% + pH < 7.35	NIV reduced re-intubation
Nava S et al., 2003 [22]	Multicenter randomized clinical trial	122	NIV ≥ 8 h/day for 48 h	Hypercapnia > 45 mmHg	NIV prevented post-extubation ventilatory failure
Ferrer M et al., 2009 [13]	Randomized clinical trial	164	NIV vs. standard oxygen	Hypercapnia > 45 mmHg	NIV reduced ventilatory failure and 90-day mortality
Girault C et al., 2011 [28]	Randomized clinical trial	388	NIV as a bridge to wean from IMV	Hypercapnia > 45 mmHg or PaCO_2_ ↑ > 10% vs. pre-extubation	NIV reduced re-intubation
Thille AW et al., 2019 [36]	Prospective observational	NR	Post-extubation HFNC or NIV	Hypercapnia > 45 mmHg	Lower re-intubation rate

Abbreviations: ABI: acute brain injury; AECOPD: acute exacerbation of chronic obstructive pulmonary disease; AHF: acute heart failure; APE: acute pulmonary edema; ARDS: acute respiratory distress syndrome; BMI: body mass index; BiPAP: bilevel positive airway pressure; COT: conventional oxygen therapy; COPD: chronic obstructive pulmonary disease; CO_2_: carbon dioxide; CPAP: continuous positive airway pressure; CPE: cardiogenic pulmonary edema; ECMO: extracorporeal membrane oxygenation; ECCO_2_R: extracorporeal carbon dioxide removal; ED: emergency department; ETCO_2_: end-tidal carbon dioxide; FiO_2_: fraction of inspired oxygen; HFNC: high-flow nasal cannula; HR: hazard ratio; ICU: intensive care unit; IMV: invasive mechanical ventilation; LOS: length of stay; NIV: non-invasive ventilation; NR: not reported; OHS: obesity hypoventilation syndrome; OSA: obstructive sleep apnea; PaCO_2_: arterial partial pressure of carbon dioxide; PaO_2_: arterial partial pressure of oxygen; PEEP: positive end-expiratory pressure; RCT: randomized controlled trial; RR: respiratory rate; SBT: spontaneous breathing trial; SD: standard deviation; SILI: self-inflicted lung injury; TBI: traumatic brain injury; Vt: tidal volume; ↑: increase; ↓: decrease.

**Table 3:** Pragmatic interpretation of PaCO_2_ in acute respiratory failure.

Clinical Context	PaCO_2_ Pattern	Main Role	Practical Message
Acute hypoxemia/pre-intubation	Low or low–normal	Prognostic/stratification	Hypocapnia may suggest higher respiratory drive and greater risk of non-invasive support failure, but it should not be used alone to determine support modality.
Cardiogenic pulmonary edema/acute heart failure/cardiogenic shock	Often low	Prognostic/monitoring	Hypocapnia has been associated with worse outcomes in some studies, but it is not, by itself, a treatment target.
Acute hypercapnic respiratory failure (COPD/OHS)	>45 mmHg, often with acidemia	Modality selection/monitoring	This is the clearest setting in which PaCO_2_ helps identify patients likely to benefit from NIV; early improvement may also support response assessment.
IMV	Hypocapnia, hypercapnia, or hypercapnic acidosis	Monitoring/prognostic	Dyscapnia is associated with outcomes, but often reflects disease severity, ventilatory strategy, or dead space rather than an independent therapeutic target.
ARDS/lung-protective ventilation	Hypercapnia may be tolerated	Contextual physiological variable	Direct correction of PaCO_2_ may conflict with lung-protective ventilation, and safe thresholds remain uncertain.
Prone positioning	Trend more important than isolated value	Response monitoring	A decrease in PaCO_2_ may support physiological response assessment, but should not be interpreted as a stand-alone treatment target.
Neurocritical care	Avoid marked hypocapnia	Safety/monitoring	PaCO_2_ is particularly important to avoid extremes, especially excessive hypocapnia.
Weaning and post-extubation	Persistent hypercapnia	Stratification/support selection	Hypercapnia may identify patients at higher risk of extubation failure and help support non-invasive strategies in selected cases.

Abbreviations: ARDS: acute respiratory distress syndrome; COPD: chronic obstructive pulmonary disease; IMV: Invasive mechanical ventilation; NIV: non-invasive ventilation; OHS: obesity hypoventilation syndrome; PaCO_2_: arterial partial pressure of carbon dioxide.

## Data Availability

No new primary data were generated in this study. All data analyzed were derived from previously published studies included in this scoping review. The decision log, PRISMA-ScR checklist, and full search strategies are openly available in the Open Science Framework (OSF) repository at https://osf.io/vszkg.

## Supplementary Materials

The following supporting information can be downloaded at https://www.mdpi.com/article/10.3390/jcm15103985/s1, Table S1. Decision log (post hoc protocol clarifications); Table S2. Preferred Reporting Items for Systematic reviews and Meta-Analyses extension for Scoping Reviews (PRISMA-ScR) Checklist; Table S3. Search strategy summary.

## References

[1] Kempker J.A., Abril M.K., Chen Y., Kramer M.R., Waller L.A., Martin G.S. (2020). The Epidemiology of Respiratory Failure in the United States 2002–2017: A Serial Cross-Sectional Study. Crit. Care Explor..

[2] Chen L., Rackley C.R. (2024). Diagnosis and Epidemiology of Acute Respiratory Failure. Crit. Care Clin..

[3] McDonald V.M., Fingleton J., Agusti A., Hiles S.A., Clark V.L., Holland A.E., Marks G.B., Bardin P.P., Beasley R., Pavord I.D. (2019). Treatable Traits: A New Paradigm for 21st Century Management of Chronic Airway Diseases: Treatable Traits Down Under International Workshop Report. Eur. Respir. J..

[4] Agusti A., Bel E., Thomas M., Vogelmeier C., Brusselle G., Holgate S., Humbert M., Jones P., Gibson P.G., Vestbo J. (2016). Treatable Traits: Toward Precision Medicine of Chronic Airway Diseases. Eur. Respir. J..

[5] Agustí A., Bafadhel M., Beasley R., Bel E.H., Faner R., Gibson P.G., Louis R., McDonald V.M., Sterk P.J., Thomas M. (2017). Precision Medicine in Airway Diseases: Moving to Clinical Practice. Eur. Respir. J..

[6] Jonkman A.H., de Vries H.J., Heunks L.M.A. (2020). Physiology of the Respiratory Drive in ICU Patients: Implications for Diagnosis and Treatment. Crit. Care.

[7] Osorio-Rodríguez E., Correa-Guerrero J., Rodelo-Barrios D., Bonilla-Llanos M., Rebolledo-Maldonado C., Patiño-Patiño J., Viera-Torres J., Arias-Gómez M., Gracia-Ordoñez M., González-Betancur D. (2025). Hypercapnia as a Double-Edged Modulator of Innate Immunity and Alveolar Epithelial Repair: A PRISMA-ScR Scoping Review. Int. J. Mol. Sci..

[8] Kato T., Kasai T., Suda S., Sato A., Ishiwata S., Yatsu S., Matsumoto H., Shitara J., Shimizu M., Murata A. (2021). Prognostic Effects of Arterial Carbon Dioxide Levels in Patients Hospitalized into the Cardiac Intensive Care Unit for Acute Heart Failure. Eur. Heart J. Acute Cardiovasc. Care.

[9] Carrillo-Aleman L., Carrasco-Gónzalez E., Araújo M.J., Guia M., Alonso-Fernández N., Renedo-Villarroya A., López-Gómez L., Higon-Cañigral A., Sanchez-Nieto J.M., Carrillo-Alcaraz A. (2022). Is Hypocapnia a Risk Factor for Non-Invasive Ventilation Failure in Cardiogenic Acute Pulmonary Edema?. J. Crit. Care.

[10] Xu X., Ma M., Min Y., Hu W., Bai L., Duan J. (2024). PaCO_2_ Is Nonlinearly Associated with NIV Failure in Patients with Hypoxemic Respiratory Failure. BMC Pulm. Med..

[11] Tiruvoipati R., Pilcher D., Buscher H., Botha J., Bailey M. (2017). Effects of Hypercapnia and Hypercapnic Acidosis on Hospital Mortality in Mechanically Ventilated Patients*. Crit. Care Med..

[12] Nin N., Muriel A., Peñuelas O., Brochard L., Lorente J.A., Ferguson N.D., Raymondos K., Ríos F., Violi D.A., Thille A.W. (2017). Severe Hypercapnia and Outcome of Mechanically Ventilated Patients with Moderate or Severe Acute Respiratory Distress Syndrome. Intensive Care Med..

[13] Ferrer M., Sellarés J., Valencia M., Carrillo A., Gonzalez G., Badia J.R., Nicolas J.M., Torres A. (2009). Non-Invasive Ventilation after Extubation in Hypercapnic Patients with Chronic Respiratory Disorders: Randomised Controlled Trial. Lancet.

[14] Algarin-Lara H., Osorio-Rodríguez E., Patiño-Patiño J., Solano-Ropero J., Rodado-Villa R. (2022). Hipercapnia Refractaria En Paciente Con Síndrome de Obesidad-Hipoventilación Maligno Y COVID-19. Reporte de Caso Y Propuesta de Manejo. Acta Colomb. Cuid. Intensivo.

[15] Peters M.D.J., Marnie C., Tricco A.C., Pollock D., Munn Z., Alexander L., McInerney P., Godfrey C.M., Khalil H. (2020). Updated Methodological Guidance for the Conduct of Scoping Reviews. JBI Evid. Synth..

[16] Tricco A.C., Lillie E., Zarin W., O’Brien K.K., Colquhoun H., Levac D., Moher D., Peters M.D.J., Horsley T., Weeks L. (2018). PRISMA Extension for Scoping Reviews (PRISMA-ScR): Checklist and Explanation. Ann. Intern. Med..

[17] Piper A.J., Sullivan C.E. (1994). Effects of Short-Term NIPPV in the Treatment of Patients With Severe Obstructive Sleep Apnea and Hypercapnia. Chest.

[18] Rabec C., Merati M., Baudouin N., Foucher P., Ulukavac T., Reybet-Degat O. (1998). Management of Obesity and Respiratory Insufficiency. The Value of Dual-Level Pressure Nasal Ventilation. Rev. Mal. Respir..

[19] Hilbert G., Gruson D., Portel L., Gbikpi-Benissan G., Cardinaud J. (1998). Noninvasive Pressure Support Ventilation in COPD Patients with Postextubation Hypercapnic Respiratory Insufficiency. Eur. Respir. J..

[20] Plant P., Owen J., Elliott M. (2000). Early Use of Non-Invasive Ventilation for Acute Exacerbations of Chronic Obstructive Pulmonary Disease on General Respiratory Wards: A Multicentre Randomised Controlled Trial. Lancet.

[21] Gattinoni L., Vagginelli F., Carlesso E., Taccone P., Conte V., Chiumello D., Valenza F., Caironi P., Pesenti A. (2003). Decrease in PaCO_2_ with Prone Position Is Predictive of Improved Outcome in Acute Respiratory Distress Syndrome*. Crit. Care Med..

[22] Nava S., Carbone G., DiBattista N., Bellone A., Baiardi P., Cosentini R., Marenco M., Giostra F., Borasi G., Groff P. (2003). Noninvasive Ventilation in Cardiogenic Pulmonary Edema. Am. J. Respir. Crit. Care Med..

[23] Díaz G.G., Alcaraz A.C., Talavera J.C.P., Pèrez P.J., Rodriguez A.E., Cordoba F.G., Hill N.S. (2005). Noninvasive Positive-Pressure Ventilation To Treat Hypercapnic Coma Secondary to Respiratory Failure. Chest.

[24] Ferrer M., Valencia M., Nicolas J.M., Bernadich O., Badia J.R., Torres A. (2006). Early Noninvasive Ventilation Averts Extubation Failure in Patients at Risk. Am. J. Respir. Crit. Care Med..

[25] González Á.O., Romero G.P.-B., Ormaechea I.F., Flores R.C., de Frutos N.C., Mangado N.G. (2006). Evolution of Patients With Chronic Obstructive Pulmonary Disease, Obesity Hypoventilation Syndrome, or Congestive Heart Failure Undergoing Noninvasive Ventilation in a Respiratory Monitoring Unit. Arch. Bronconeumol. (Engl. Ed.).

[26] Duarte A.G., Justino E., Bigler T., Grady J. (2007). Outcomes of Morbidly Obese Patients Requiring Mechanical Ventilation for Acute Respiratory Failure*. Crit. Care Med..

[27] Sellares J., Ferrer M., Cano E., Loureiro H., Valencia M., Torres A. (2011). Predictors of Prolonged Weaning and Survival during Ventilator Weaning in a Respiratory ICU. Intensive Care Med..

[28] Girault C., Bubenheim M., Abroug F., Diehl J.L., Elatrous S., Beuret P., Richecoeur J., L’Her E., Hilbert G., Capellier G. (2011). Noninvasive Ventilation and Weaning in Patients with Chronic Hypercapnic Respiratory Failure. Am. J. Respir. Crit. Care Med..

[29] Carrillo A., Ferrer M., Gonzalez-Diaz G., Lopez-Martinez A., Llamas N., Alcazar M., Capilla L., Torres A. (2012). Noninvasive Ventilation in Acute Hypercapnic Respiratory Failure Caused by Obesity Hypoventilation Syndrome and Chronic Obstructive Pulmonary Disease. Am. J. Respir. Crit. Care Med..

[30] Marik P.E., Desai H. (2013). Characteristics of Patients With the “Malignant Obesity Hypoventilation Syndrome” Admitted to an ICU. J. Intensive Care Med..

[31] Pu L., Zhu B., Jiang L., Du B., Zhu X., Li A., Li G., He Z., Chen W., Ma P. (2015). Weaning Critically Ill Patients from Mechanical Ventilation: A Prospective Cohort Study. J. Crit. Care.

[32] Çiftci F., Çiledağ A., Erol S., Öz M., Acar D., Kaya A. (2017). Evaluation of the Feasibility of Average Volume-Assured Pressure Support Ventilation in the Treatment of Acute Hypercapnic Respiratory Failure Associated with Chronic Obstructive Pulmonary Disease: A Pilot Study. J. Crit. Care.

[33] Fuller B.M., Mohr N.M., Drewry A.M., Ferguson I.T., Trzeciak S., Kollef M.H., Roberts B.W. (2017). Partial Pressure of Arterial Carbon Dioxide and Survival to Hospital Discharge among Patients Requiring Acute Mechanical Ventilation: A Cohort Study. J. Crit. Care.

[34] Sellares J., Ferrer M., Anton A., Loureiro H., Bencosme C., Alonso R., Martinez-Olondris P., Sayas J., Peñacoba P., Torres A. (2017). Discontinuing Noninvasive Ventilation in Severe Chronic Obstructive Pulmonary Disease Exacerbations: A Randomised Controlled Trial. Eur. Respir. J..

[35] Bry C., Jaffré S., Guyomarc’h B., Corne F., Chollet S., Magnan A., Blanc F.-X. (2018). Noninvasive Ventilation in Obese Subjects After Acute Respiratory Failure. Respir. Care.

[36] Thille A.W., Muller G., Gacouin A., Coudroy R., Decavèle M., Sonneville R., Beloncle F., Girault C., Dangers L., Lautrette A. (2019). Effect of Postextubation High-Flow Nasal Oxygen With Noninvasive Ventilation vs High-Flow Nasal Oxygen Alone on Reintubation Among Patients at High Risk of Extubation Failure. JAMA.

[37] Li X.-Y., Tang X., Wang R., Yuan X., Zhao Y., Wang L., Li H.-C., Chu H.-W., Li J., Mao W.-P. (2020). High-Flow Nasal Cannula for Chronic Obstructive Pulmonary Disease with Acute Compensated Hypercapnic Respiratory Failure: A Randomized, Controlled Trial. Int. J. Chron. Obstruct. Pulmon. Dis..

[38] Madotto F., Rezoagli E., McNicholas B.A., Pham T., Slutsky A.S., Bellani G., Laffey J.G. (2020). Patterns and Impact of Arterial CO_2_ Management in Patients with Acute Respiratory Distress Syndrome. Chest.

[39] Tsonas A.M., Botta M., Horn J., Morales-Quinteros L., Artigas A., Schultz M.J., Paulus F., Neto A.S. (2022). Clinical Characteristics, Physiological Features, and Outcomes Associated with Hypercapnia in Patients with Acute Hypoxemic Respiratory Failure due to COVID–19—Insights from the PRoVENT–COVID Study. J. Crit. Care.

[40] Braunsteiner J., Castro L., Wiessner C., Grensemann J., Schroeder M., Burdelski C., Sensen B., Kluge S., Fischer M. (2024). Association Between Dyscapnia, Ventilatory Variables, and Mortality in Patients With Acute Respiratory Distress Syndrome—A Retrospective Cohort Study. J. Intensive Care Med..

[41] Robba C., Battaglini D., Abbas A., Sarrió E., Cinotti R., Asehnoune K., Taccone F.S., Rocco P.R., Schultz M.J., Citerio G. (2024). Clinical Practice and Effect of Carbon Dioxide on Outcomes in Mechanically Ventilated Acute Brain-Injured Patients: A Secondary Analysis of the ENIO Study. Intensive Care Med..

[42] Tan D., Wang B., Cao P., Wang Y., Sun J., Geng P., Walline J.H., Wang Y., Wang C. (2024). High Flow Nasal Cannula Oxygen Therapy versus Non-Invasive Ventilation for Acute Exacerbations of Chronic Obstructive Pulmonary Disease with Acute-Moderate Hypercapnic Respiratory Failure: A Randomized Controlled Non-Inferiority Trial. Crit. Care.

[43] Villar J., González-Martín J.M., Fernández C., Soler J.A., Ambrós A., Pita-García L., Fernández L., Ferrando C., Arocas B., González-Vaquero M. (2024). Predicting the Length of Mechanical Ventilation in Acute Respiratory Disease Syndrome Using Machine Learning: The PIONEER Study. J. Clin. Med..

[44] Rusnak J., Schupp T., Weidner K., Ruka M., Egner-Walter S., Schmitt A., Akin M., Mashayekhi K., Ayoub M., Behnes M. (2025). Partial Arterial Carbon Dioxide and Oxygen Pressure in Patients with Cardiogenic Shock. Intern. Emerg. Med..

[45] Brochard L., Slutsky A., Pesenti A. (2017). Mechanical Ventilation to Minimize Progression of Lung Injury in Acute Respiratory Failure. Am. J. Respir. Crit. Care Med..

[46] Helms J., Catoire P., Abensur Vuillaume L., Bannelier H., Douillet D., Dupuis C., Federici L., Jezequel M., Jozwiak M., Kuteifan K. (2024). Oxygen Therapy in Acute Hypoxemic Respiratory Failure: Guidelines from the SRLF-SFMU Consensus Conference. Ann. Intensive Care.

[47] Seow D., Khor Y.H., Khung S.-W., Smallwood D.M., Ng Y., Pascoe A., Smallwood N. (2024). High-Flow Nasal Oxygen Therapy Compared with Conventional Oxygen Therapy in Hospitalised Patients with Respiratory Illness: A Systematic Review and Meta-Analysis. BMJ Open Respir. Res..

[48] Marjanovic N., Piton M., Lamarre J., Alleyrat C., Couvreur R., Guenezan J., Mimoz O., Frat J.-P. (2024). High-Flow Nasal Cannula Oxygen versus Noninvasive Ventilation for the Management of Acute Cardiogenic Pulmonary Edema: A Randomized Controlled Pilot Study. Eur. J. Emerg. Med..

[49] Marjanovic N., Couvreur R., Lamarre J., Piton M., Guenezan J., Mimoz O. (2024). High-Flow Nasal Cannula Oxygen Therapy versus Noninvasive Ventilation in Acute Respiratory Failure Related to Suspected or Confirmed Acute Heart Failure: A Systematic Review with Meta-Analysis. Eur. J. Emerg. Med..

[50] Masip J., Peacock W.F., Price S., Cullen L., Martin-Sanchez F.J., Seferovic P., Maisel A.S., Miro O., Filippatos G., Vrints C. (2018). Indications and Practical Approach to Non-Invasive Ventilation in Acute Heart Failure. Eur. Heart J..

[51] Berbenetz N., Wang Y., Brown J., Godfrey C., Ahmad M., Vital F.M., Lambiase P., Banerjee A., Bakhai A., Chong M. (2019). Non-Invasive Positive Pressure Ventilation (CPAP or Bilevel NPPV) for Cardiogenic Pulmonary Oedema. Cochrane Database Syst. Rev..

[52] Rochwerg B., Brochard L., Elliott M.W., Hess D., Hill N.S., Nava S., Navalesi P., Antonelli M., Brozek J., Conti G. (2017). Official ERS/ATS Clinical Practice Guidelines: Noninvasive Ventilation for Acute Respiratory Failure. Eur. Respir. J..

[53] Carteaux G., Millán-Guilarte T., De Prost N., Razazi K., Abid S., Thille A.W., Schortgen F., Brochard L., Brun-Buisson C., Mekontso Dessap A. (2016). Failure of Noninvasive Ventilation for De Novo Acute Hypoxemic Respiratory Failure. Crit. Care Med..

[54] Matthay M.A. (2015). Saving Lives with High-Flow Nasal Oxygen. N. Engl. J. Med..

[55] Yoshida T., Uchiyama A., Matsuura N., Mashimo T., Fujino Y. (2012). Spontaneous Breathing during Lung-Protective Ventilation in an Experimental Acute Lung Injury Model. Crit. Care Med..

[56] Osadnik C.R., Tee V.S., Carson-Chahhoud K.V., Picot J., Wedzicha J.A., Smith B.J. (2017). Non-Invasive Ventilation for the Management of Acute Hypercapnic Respiratory Failure due to Exacerbation of Chronic Obstructive Pulmonary Disease. Cochrane Database Syst. Rev..

[57] Contou D., Fragnoli C., Córdoba-Izquierdo A., Boissier F., Brun-Buisson C., Thille A.W. (2015). Severe but Not Mild Hypercapnia Affects the Outcome in Patients with Severe Cardiogenic Pulmonary Edema Treated by Non-Invasive Ventilation. Ann. Intensive Care.

[58] Masa J.F., Utrabo I., Gomez de Terreros J., Aburto M., Esteban C., Prats E., Núñez B., Ortega-González Á., Jara-Palomares L., Martin-Vicente M.J. (2016). Noninvasive Ventilation for Severely Acidotic Patients in Respiratory Intermediate Care Units. BMC Pulm. Med..

[59] Scala R., Naldi M., Archinucci I., Coniglio G., Nava S. (2005). Noninvasive Positive Pressure Ventilation in Patients With Acute Exacerbations of COPD and Varying Levels of Consciousness. Chest.

[60] Xu C., Yang F., Wang Q., Gao W. (2023). Comparison of High Flow Nasal Therapy with Non-Invasive Ventilation and Conventional Oxygen Therapy for Acute Hypercapnic Respiratory Failure: A Meta-Analysis of Randomized Controlled Trials. Int. J. Chron. Obstruct. Pulmon. Dis..

[61] Gendreau S., Geri G., Pham T., Vieillard-Baron A., Mekontso Dessap A. (2022). The Role of Acute Hypercapnia on Mortality and Short-Term Physiology in Patients Mechanically Ventilated for ARDS: A Systematic Review and Meta-Analysis. Intensive Care Med..

[62] Serpa Neto A., Deliberato R.O., Johnson A.E.W., Bos L.D., Amorim P., Pereira S.M., Cazati D.C., Cordioli R.L., Correa T.D., Pollard T.J. (2018). Mechanical Power of Ventilation Is Associated with Mortality in Critically Ill Patients: An Analysis of Patients in Two Observational Cohorts. Intensive Care Med..

[63] Coppola S., Caccioppola A., Froio S., Formenti P., De Giorgis V., Galanti V., Consonni D., Chiumello D. (2020). Effect of Mechanical Power on Intensive Care Mortality in ARDS Patients. Crit. Care.

[64] Monteiro A.C.C., Vangala S., Wick K.D., Delucchi K.L., Siegel E.R., Thompson B.T., Liu K.D., Sapru A., Sinha P., Matthay M.A. (2022). The Prognostic Value of Early Measures of the Ventilatory Ratio in the ARDS ROSE Trial. Crit. Care.

[65] Carney N., Totten A.M., O’Reilly C., Ullman J.S., Hawryluk G.W.J., Bell M.J., Bratton S.L., Chesnut R., Harris O.A., Kissoon N. (2017). Guidelines for the Management of Severe Traumatic Brain Injury, Fourth Edition. Neurosurgery.

[66] Grasselli G., Calfee C.S., Camporota L., Poole D., Amato M.B.P., Antonelli M., Arabi Y.M., Baroncelli F., Beitler J.R., Bellani G. (2023). ESICM Guidelines on Acute Respiratory Distress Syndrome: Definition, Phenotyping and Respiratory Support Strategies. Intensive Care Med..

[67] Qadir N., Sahetya S., Munshi L., Summers C., Abrams D., Beitler J., Bellani G., Brower R.G., Burry L., Chen J.-T. (2024). An Update on Management of Adult Patients with Acute Respiratory Distress Syndrome: An Official American Thoracic Society Clinical Practice Guideline. Am. J. Respir. Crit. Care Med..

[68] Charron C., Repesse X., Bouferrache K., Bodson L., Castro S., Page B., Jardin F., Vieillard-Baron A. (2011). PaCO_2_ and Alveolar Dead Space Are More Relevant than PaO_2_/FiO_2_ Ratio in Monitoring the Respiratory Response to Prone Position in ARDS Patients: A Physiological Study. Crit. Care.

[69] Nava S., Gregoretti C., Fanfulla F., Squadrone E., Grassi M., Carlucci A., Beltrame F., Navalesi P. (2005). Noninvasive Ventilation to Prevent Respiratory Failure after Extubation in High-Risk Patients*. Crit. Care Med..

[70] McNamee J.J., Gillies M.A., Barrett N.A., Perkins G.D., Tunnicliffe W., Young D., Bentley A., Harrison D.A., Brodie D., Boyle A.J. (2021). Effect of Lower Tidal Volume Ventilation Facilitated by Extracorporeal Carbon Dioxide Removal vs Standard Care Ventilation on 90-Day Mortality in Patients with Acute Hypoxemic Respiratory Failure. JAMA.

[71] Zhou Z., Li Z., Liu C., Wang F., Zhang L., Fu P. (2023). Extracorporeal Carbon Dioxide Removal for Patients with Acute Respiratory Failure: A Systematic Review and Meta-Analysis. Ann. Med..

[72] Stommel A.-M., Herkner H., Kienbacher C.L., Wildner B., Hermann A., Staudinger T. (2024). Effects of Extracorporeal CO2 Removal on Gas Exchange and Ventilator Settings: A Systematic Review and Meta-Analysis. Crit. Care.

